# Pyrazinamide triggers degradation of its target aspartate decarboxylase

**DOI:** 10.1038/s41467-020-15516-1

**Published:** 2020-04-03

**Authors:** Pooja Gopal, Jickky Palmae Sarathy, Michelle Yee, Priya Ragunathan, Joon Shin, Shashi Bhushan, Junhao Zhu, Tatos Akopian, Olga Kandror, Teck Kwang Lim, Martin Gengenbacher, Qingsong Lin, Eric J. Rubin, Gerhard Grüber, Thomas Dick

**Affiliations:** 1 Department of Medicine, Yong Loo Lin School of Medicine, National University of Singapore, Singapore, Singapore; 2 Department of Microbiology and Immunology, Yong Loo Lin School of Medicine, National University of Singapore, Singapore, Singapore; 3 School of Biological Sciences, Nanyang Technological University, Singapore, Singapore; 4 Department of Immunology and Infectious Diseases, Harvard T.H. Chan School of Public Health, Harvard University, Boston, MA USA; 5 Department of Biological Sciences, National University of Singapore, Singapore, Singapore; 6 Center for Discovery and Innovation, Hackensack Meridian Health, Nutley, NJ USA; 7 Department of Medical Sciences, Hackensack Meridian Medical School at Seton Hall University, Nutley, NJ USA; 8 Present Address: MSD Translational Medicine Research Centre, Merck Research Laboratories, Singapore, Singapore

**Keywords:** Target identification, Tuberculosis

## Abstract

Pyrazinamide is a sterilizing first-line tuberculosis drug. Genetic, metabolomic and biophysical analyses previously demonstrated that pyrazinoic acid, the bioactive form of the prodrug pyrazinamide (PZA), interrupts biosynthesis of coenzyme A in *Mycobacterium tuberculosis* by binding to aspartate decarboxylase PanD. While most drugs act by inhibiting protein function upon target binding, we find here that pyrazinoic acid is only a weak enzyme inhibitor. We show that binding of pyrazinoic acid to PanD triggers degradation of the protein by the caseinolytic protease ClpC1-ClpP. Thus, the old tuberculosis drug pyrazinamide exerts antibacterial activity by acting as a target degrader, a mechanism of action that has recently emerged as a successful strategy in drug discovery across disease indications. Our findings provide the basis for the rational discovery of next generation PZA.

## Abstract (web summary)

It has been shown that the bioactive component of pyrazinamide, pyrazinoic acid (POA), blocks coenzyme A biosynthesis in *M. tuberculosis* by binding to the aspartate decarboxylase PanD. Here the authors show that pyrazinamide triggers degradation of PanD by stimulating its degradation by the caseinolytic protease Clp.

## Introduction

The introduction of pyrazinamide (PZA) in the tuberculosis drug regimen shortened treatment from 12 to 6 months^1^. PZA is a prodrug that is activated by a *Mycobacterium tuberculosis* (Mtb) amidase to release its bioactive component pyrazinoic acid (POA)^2^. Aspartate decarboxylase PanD, required for Coenzyme A (CoA) biosynthesis, emerged as a target of POA^3–6^. In vitro and in vivo screening to isolate spontaneous POA-resistant Mtb mutants identified missense mutations in either *panD* or the unfoldase *clpC1*, encoding a component of the caseinolytic protease ClpC1–ClpP^3,5–8^. Overexpression and binding studies with PanD or ClpC1 pointed to PanD as the direct target of POA, whereas *clpC1* mutations appeared to indirectly cause resistance^3,4,6,8,9^. Indeed, supplementing growth media with CoA precursors downstream of the PanD-catalyzed step conferred POA resistance^3,6,10^. Metabolomic analyses confirmed inhibition of PanD by POA^4^ and biophysical studies using recombinant proteins showed that PanD missense mutations found in POA-resistant strains prevented POA binding^4^. Together, these results established PanD and the CoA biosynthetic pathway as a target of POA^3,4,6,10^.

Classically, antibacterials act by inhibiting the function of their target. To characterize POA’s on-target activity, we measured the inhibitory effect of the drug on the enzymatic conversion of aspartate to β-alanine by PanD. Surprisingly, we only observed a weak effect at extremely high concentrations (see “Results”). If POA does not effectively inhibit the catalytic activity of PanD, how does it block the PanD-catalyzed step in CoA synthesis? Interestingly, mutations in PanD and ClpC1 cause the same level of PZA/POA resistance in Mtb^7^. This suggests a mechanistic link between these two proteins and POA. The unfoldase ClpC1 is part of the caseinolytic protease complex ClpC1–ClpP, involved in the degradation of substrate proteins^11,12^. In ClpC1, POA resistance mutations are found primarily in the N-terminal and middle domains, proposed to affect substrate selectivity of the complex^7,9^. Thus, we speculated that PanD may be recognized and degraded by this machinery. ClpC1–ClpP recognizes substrates via short C-terminal tags^11–13^. Curiously, the mycobacterial PanD protein contains a 13 amino acid C-terminal extension of unknown function^14^. Mutations in this tail cause POA resistance and prevent drug binding^3–6^. Therefore, we hypothesized that (1) the C-terminal tail of Mtb PanD constitutes a degradation tag that is recognized by ClpC1–ClpP and (2) binding of POA to PanD triggers increased degradation of the target. Here, we tested these two hypotheses and show that PanD’s C-terminal tail indeed constitutes a degradation tag and that POA binding to PanD stimulates degradation of the protein by ClpC1–ClpP. Thus, the tuberculosis drug PZA promotes degradation of its target. While not yet reported for an antibacterial, drug-induced target degradation has recently emerged as a strategy in drug discovery for other disease indications.

## Results

### POA is only a weak PanD enzyme inhibitor

**Fig. 1:**
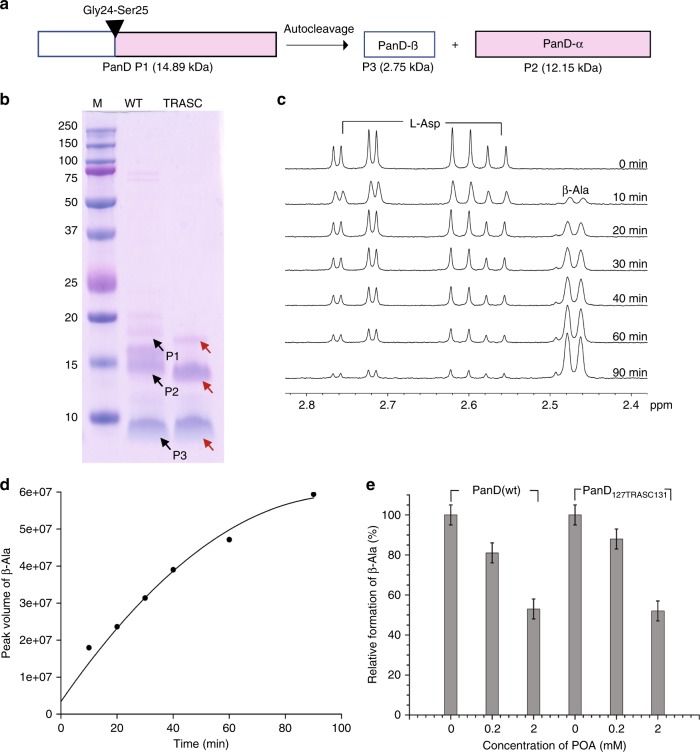
Verification of purified recombinant PanD proteins and effect of POA on PanD enzymatic activity. **a** Schematic depicting the autocleavage of PanD at the Gly24-Ser25 position to yield enzymatically active enzyme comprising of a C-terminal (PanD-α) and an N-terminal fragment (PanD-β)^15^. **b** Twelve percent SDS-PAGE of purified, N-terminal His-tagged recombinant proteins PanD_WT_ (WT) and C-terminal truncated, non-POA binding PanD_127TRASC131_ (TRASC). To determine the identity of the protein bands, the bands corresponding to P1, P2, and P3 were cut out, in-gel digested with trypsin and subjected to LC–MS analysis. PanD is synthesized as a proenzyme and rapidly autocatalytically cleaved at its N-terminus, resulting in a small N-terminal fragment (P3, expected size including His Tag for WT and TRASC: 3.75 kDa) and a larger C-terminal polypeptide (P2, expected size for WT: 12.15 kDa and TRASC: 11.4 kDa). Traces of uncleaved proenzyme are indicated (P1, expected size including His Tag for WT: 15.9 kDa and TRASC: 15.15 kDa). Black and red arrows indicate the corresponding protein bands of wild-type and truncated PanD, respectively. **c**, **d** Time-dependent conversion of L-Aspartate (2 mM) to β-Alanine (β-Ala) as determined by ^1^H NMR after addition of 10 µM Mtb PanD_WT_ in D_2_O at 298 K on a Bruker Avance 400 MHz spectrometer. Shown are (**c**) representative ^1^H NMR spectra and (**d**) a plot of time-dependent β-Alanine formation at different incubation times by measuring the peak volume of converted L-Asp to β-Ala. At *t* = 40 min, ~50% conversion of L-aspartate to β-Alanine was observed as determined by integration of peak values. Thus, 40 min was chosen as a reference point within the linear range of PanD enzyme kinetics for subsequent enzyme assays. **e** Effect of POA on the formation of β-Ala by recombinant PanD_WT_ and POA-resistant PanD_127TRASC131_ proteins, respectively, as determined by ^1^H NMR. The plot shows the results of a representative experiment. NMR spectra for **e** are shown in Supplementary Fig. 1. Both recombinant proteins were purified as described^4^. The NMR experiments were repeated twice independently, yielding the same results. The results from a representative experiment are shown. Error bars were defined as 5% standard deviations. Source data are provided as a Source Data file. The cleaved form of the wild-type protein P2 migrates as a doublet with similar staining intensity. The reason for this behavior remains to be determined. LC–MS analysis of the doublet band suggests that both sub-bands present P2 (Supplementary Data 1_P123seq). The P2 doublet is specific to recombinant wild-type protein and is not observed in the mutant PanD_127TRASC131_ or in whole cell extracts (see Fig. 4, western blot analyses). The SDS-PAGE analyses show that the recombinant PanD protein preparations used for in vitro analyses in the current study presented largely the cleaved, active form of the enzyme.

The aspartate decarboxylase PanD is a proenzyme activated by autocatalytic cleavage (Fig. 1a, ref. ^15^). To characterize POA’s on-target activity we purified recombinant PanD in its cleaved active form (Fig. 1b) and measured the inhibitory effect of POA on the enzymatic conversion of aspartate to β-alanine. Surprisingly, we only observed a weak effect at high concentrations. Although POA binds PanD with a dissociation constant *K*_D_ of 6^4^, 200 µM POA reduced β-alanine production by only 15% (Fig. 1e). Even “bathing” the enzyme in 2 mM POA reduced product formation by only 47% (Fig. 1e). These weak effects were not significantly attenuated when a POA-resistant PanD mutant that does not bind POA was used (Fig. 1e). This suggests that POA is not an effective inhibitor of PanD enzymatic activity.

### PanD is substrate of caseinolytic protease

**Fig. 2:**
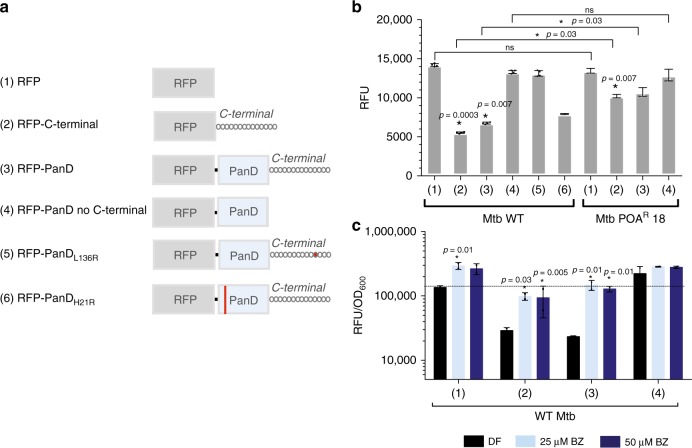
Effect of fusion of various PanD derivatives on the level of RFP reporter protein. **a** Schematic of derivatives of PanD fused translationally to constitutively expressed red fluorescence protein (RFP) as episomal reporter on plasmid pMV262 and transformed into Mtb. C-terminal = mycobacterium-specific C-terminal tail of PanD. RFP–PanD_L136R_ and RFP–PanD_H21R_ are PZA/POA-resistant mutants harboring missense mutation in the N-terminal His21 or the C-terminal tail Leu136 (indicated in red) and have been described previously^4,6^. **b** Fluorescence levels (expressed as RFU, relative fluorescence units) of mid-log phase cultures (OD_600_ = 0.2) were determined as a measure of intrabacterial fusion protein level in either Mtb wild-type (WT) or PZA/POA-resistant mutant Mtb POA^R^18 [*clpC1*: Lys209Glu]^7^ harboring RFP–PanD reporter constructs shown in **a**. One-way ANOVA multiple comparisons test (Dunn’s posttest, GraphPad Prism) was used to compare fluorescence levels conferred by the various constructs relative to the native RFP in wild-type background (left cluster 1–6) and in *clpC1* mutant background (right cluster 1–4), respectively; *p value < 0.05; ^ns^*p* value > 0.05. The two-sided Mann–Whitney test (GraphPad Prism) was used to compare fluorescence levels conferred by each RFP construct in the *clpC1* mutant versus wild-type background (horizontal bars placed across left and right clusters). *p value < 0.05; ^ns^*p* value > 0.05. **c** Effect of bortezomib (BZ) on fluorescence of Mtb cultures carrying different RFP–PanD reporter constructs shown in **a**. Cultures were treated with bortezomib (Mtb MIC_50_ = 25 µM) for 3 days. One-way ANOVA multiple comparisons and Dunn’s posttest (GraphPad Prism) were used to compare fluorescence levels in drug-free versus BZ-treated cultures. *p value < 0.05. The dotted line indicates mean normalized culture fluorescence of drug-free RFP controls. Experiments were repeated twice independently with two technical replicates. Error bars were defined as standard deviations. Source data are provided as a Source Data file.

To test the hypothesis that the C-terminal tail of Mtb PanD constitutes a degradation tag that is recognized by ClpC1–ClpP, we constructed red fluorescent protein (RFP) C-terminal reporter fusions^13^ and measured the effect of various PanD modifications on protein levels in Mtb (Fig. 2a). As expected, the expression of native RFP alone showed a high fluorescence baseline (Fig. 2b). Attachment of PanD’s 13 amino acid C-terminal tail to RFP caused a reduction in fluorescence, indicating that the tail acts as a degradation signal. The same effect was observed when full-length PanD was fused to RFP. Consistent with the C-terminal tail acting as a degradation tag, fusion of PanD lacking its native C-terminal tail to RFP restored baseline fluorescence levels. Similar fluorescence was also observed for a fusion carrying a missense mutation in the C-terminal tail^6^, consistent with amino acid-sequence recognition by the degradation machinery (Fig. 2b). These experiments were repeated independently in *M. bovis* BCG, yielding the same results (Supplementary Fig. 2). To determine whether ClpC1 is involved in degradation of PanD’s C-terminal tail, we measured the effect of introducing each RFP fusion into a POA-resistant *clpC1* mutant strain (Fig. 2b). The level of native RFP was not affected by the *clpC1* mutation compared with wild-type *clpC1*, and baseline fluorescence levels were observed in both genetic backgrounds. As hypothesized, RFP fusions containing the C-terminal tail alone or full-length PanD showed increased fluorescence levels in *clpC1* mutant background, indicating impaired degradation activity by the POA-resistant *clpC1* mutant. These results suggested that PanD’s C-terminal degradation tag is recognized by ClpC1, which led us to postulate that PanD degradation is mediated by the caseinolytic protease ClpP. As *clpP* is genetically essential and cannot be deleted, we employed a pharmacological approach. Treatment of Mtb RFP fusion strains with bortezomib, a mycobacterial ClpP inhibitor^16^, increased fluorescence of cultures containing RFP-C-terminal tail as well as RFP-full-length PanD fusions to the baseline levels of native RFP (Fig. 2c). We further confirmed ClpC1–ClpP mediated recognition and degradation of PanD by in vitro reconstitution of the process using recombinant ClpC1 and ClpP proteins^17^, and cell-free synthesized N-terminally eGFP-tagged PanD as substrate (Supplementary Fig. 3). Taken together, these data demonstrate that PanD is a substrate of the caseinolytic protease complex ClpC1–ClpP and that the mycobacterium-specific tail of PanD is a tag recognized by the protease complex. Our finding is consistent with a previous screen for substrates of ClpP, in which Raju et al. identified PanD as one of the 132 Mtb proteins overrepresented upon conditional depletion of Mtb ClpP^12^.

### POA stimulates PanD degradation via caseinolytic protease

**Fig. 3:**
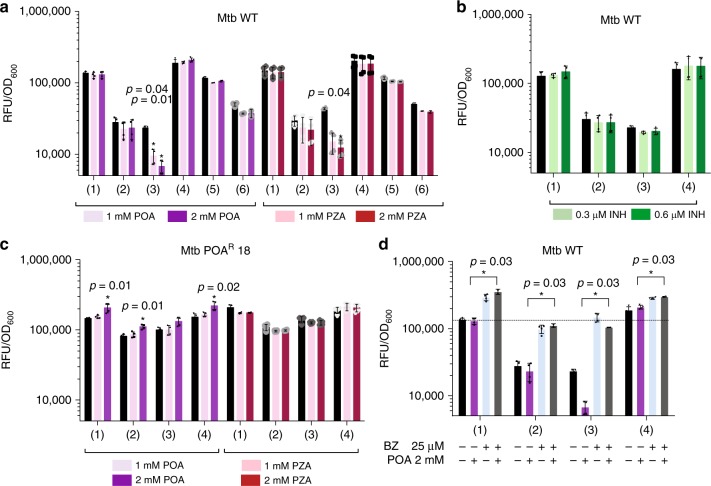
Effect of POA, PZA, INH treatment, and POA/bortezomib co-treatment on levels of various RFP–PanD fusions. Mtb strains harboring various RFP–PanD fusions shown in Fig. 2a were treated with increasing doses of POA, bortezomib (BZ), and isoniazid as control drug (INH) for 3 days. Fluorescence (RFU) was measured and normalized to bacterial growth (OD_600_). **a** RFP–PanD fusions in Mtb wild type (WT) treated with POA (1 mM or Mtb MIC_50_) and PZA. **b** RFP–PanD fusions in Mtb wild type treated with isoniazid (INH): 0.3 µM or Mtb MIC_50_. **c** RFP–PanD fusions in POA-resistant *clpC1* mutant Mtb POA^R^18 [*clpC1*: Lys209Glu] treated with POA and PZA. **d** RFP–PanD fusions in Mtb wild type treated with BZ (25 µM or Mtb MIC_50_) or POA (2 mM) alone or in combination as indicated. The dashed line indicates mean normalized culture fluorescence of drug-free RFP controls. **a**–**c** One-way ANOVA multiple comparisons test (Dunn’s posttest, GraphPad Prism) was used to compare fluorescence levels in drug-treated versus drug-free controls. *p value < 0.05. **d** The two-sided Mann–Whitney *U* test (GraphPad Prism) was used to compare the effect of POA treatment on fluorescence levels conferred by RFP constructs 1–4 (**a** wild-type background) in the presence and absence of BZ. *p value < 0.05. For each strain and treatment, drug-free controls were included (black bars). Experiments were repeated twice independently with two technical replicates. Error bars were defined as standard deviations. Source data are provided as a Source Data file.

**Fig. 4:**
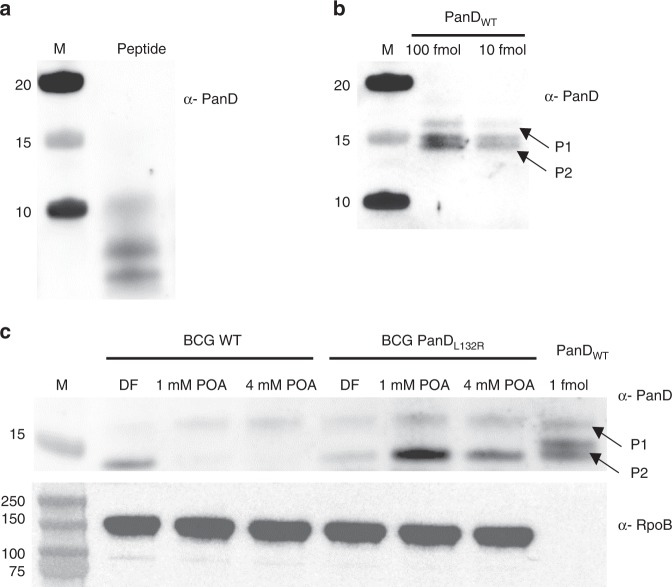
Effect of POA treatment on PanD protein levels in wild-type and POA-resistant *panD*-mutant *M. bovis* BCG. **a**, **b** Western blots of 1 µg PanD peptide IAYATMDDARARTY used as antigen, and 100 and 10 fmol purified recombinant PanD wild-type protein (PanD_WT_), respectively. P2, cleaved C-terminal portion of PanD; P1 full-length proenzyme of PanD (see Fig. 1). Antiserum α-PanD against a PanD peptide was raised in rabbits and used to analyze protein levels of native PanD protein by western blotting. The cleaved N-terminal part of PanD (P3, Fig. 1) is not visible on the blot as the PanD peptide used to raise the antiserum is located within the P2 portion of the protein. As observed in Coomassie blue stained SDS-PAGE of purified recombinant PanD protein (Fig. 1), the cleaved form of P2 migrates as a doublet with similar staining intensity. The P2 doublet is specific to recombinant protein and is not observed in whole cell extracts (see **c**). **c** *M. bovis* BCG wild type (BCG WT) and POA-resistant *M. bovis* BCG PanD_L132R_ harboring a Leu132Arg mutation in its C-terminal tail^6^ were either not treated with POA (DF drug free) or treated with 1 or 4 mM POA for 24 h. Ten micrograms of respective total protein extracts was subjected to western blot analyses. Upper panel: probing with α-PanD. PanD_WT_, 1 fmol of recombinant PanD wild-type protein was included as molecular weight marker. Lower panel: probing of the blot showed in the upper panel with antiserum against mycobacterial RNA polymerase subunit RpoB (α-RpoB) to show equal loading. M, Marker with molecular weights (kilodalton) are indicated. Experiments were repeated two times independently, yielding the same results. The results from a representative experiment are shown. Taken together, these data show that treatment with POA causes a reduction of the intrabacterial level of wild-type PanD but does not affect the intrabacterial level of a non-POA binding resistant mutant of PanD. Consistent with the overall low intrabacterial levels of PanD indicated by our western blotting experiments, PanD was not detectable in previous proteome analyses (<1 fmol/µg), whereas all other CoA pathway enzymes could be quantified (1–6 fmol/µg)^29^. We confirmed this finding by shotgun proteomic analyses of mycobacterial whole cell extracts in which CoA pathway enzymes other than PanD were readily detectable, including the pantothenate synthetase PanC (Supplementary Data 2_Proteomic analyses).

Having established that PanD’s C-terminal tail is a degradation tag and PanD levels are regulated posttranslationally, we asked whether POA treatment enhances PanD degradation. POA treatment of cultures harboring the native RFP did not affect fluorescence (Fig. 3a), neither did it affect fluorescence of cultures expressing the RFP-C-terminal fusion, consistent with the requirement of a dual epitope (including His21, Fig. 2a) for POA binding to PanD^4^. In contrast, the full-length PanD fusion showed a dose-dependent reduction of fluorescence by POA. This reduction was dependent on PanD’s C-terminal tail and was not observed in cultures harboring fusion with POA-resistant PanD proteins containing missense mutations in either the N-terminal His21 or the C-terminal tail, both of which abolishing binding of POA (Fig. 3a, ref. ^4^). To confirm that POA indeed causes reduction of endogenous PanD levels, we performed western blotting with an anti-PanD antibody, and showed rapid reduction of native PanD in response to POA treatment in wild-type *M. bovis* BCG but not in a POA-resistant C-terminal PanD missense mutant (Fig. 4 and Supplementary Fig. 4). Further, RFP reporter-based work showed that increased degradation of PanD in Mtb was also observed upon treatment with PZA, the prodrug of POA (Fig. 3a), but not with the control drug isoniazid (Fig. 3b), that targets mycolic acid synthesis. This showed that increased PanD degradation is POA/PZA-specific and not a general drug treatment effect. PanD mRNA levels were not affected by POA treatment, ruling out transcriptional effects (Supplementary Fig. 5). To determine whether ClpC1 is involved in POA’s mechanism of action, we tested the impact of *clpC1* mutation on POA-induced PanD degradation by measuring the effect of POA on the various RFP–PanD fusions in the *clpC1* mutant background. POA-resistant *clpC1* mutations prevented POA/PZA-induced reduction of PanD levels (Fig. 3c). To determine whether the ClpP protease is required for POA-induced PanD degradation, we co-treated cultures with POA and the ClpP inhibitor bortezomib. Inhibition of ClpP prevented POA-induced reduction of PanD levels (Fig. 3d). It is to note that bortezomib also increased fluorescence of the RFP control strain, although to a lesser extent than the PanD-RFP reporter strains. The reasons for this effect remain to be investigated and may include yet unidentified off-target effects. To determine whether the other major protein degradation machinery of Mtb, the proteasome^18^, contributes to PanD degradation, we measured the effect of *prcAB* gene deletion on POA susceptibility. POA MIC was not affected by the deletion, arguing against proteasome involvement in PanD degradation (Supplementary Fig. 6). Together, these results suggest that the mechanism of action of PZA/POA involves suicidal derailing of a bacterial posttranslational regulatory mechanism in which POA promotes the breakdown of its essential target by Mtb.

### POA changes quaternary and secondary structures of PanD

**Fig. 5:**
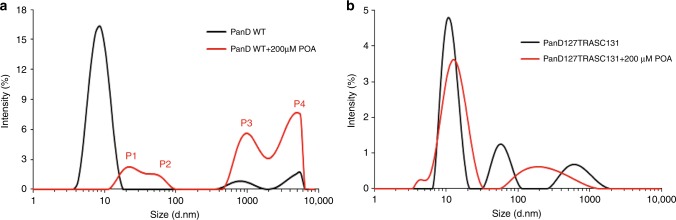
Effect of POA on PanD_WT_ and resistant mutant, non-POA binding PanD_127TRASC131_ as determined by dynamic light scattering. **a** The distribution of the intensity of dynamic light scattering by PanD_WT_ without drug (black) and PanD_WT_ with 200 µM POA (red) as a function of particle diameter (*d* in nm) shows that PanD_WT_ transforms into higher oligomers (P1–4) upon addition of POA with calculated hydrodynamic diameters of 21.04 ± 7.22, 55.04 ± 6.35, 955.4 ± 106.9, and 4801 ± 1134 nm, respectively. The estimated intensities of peaks 1–4 were 11%, 8%, 38%, and 43%, respectively. **b** In contrast, the PanD_127TRASC131_ mutant retained its prominent peak at around 11.5 nm in the presence (red) of 200 μM POA (drug-free control: black). Experiments were repeated three times yielding the same results. The results from a representative experiment are shown. These data show that POA causes oligomer formation of wild-type PanD but not of a POA-resistant mutant version of the protein. These results suggest that the drug does not cause protein aggregation per se. Source data are provided as a Source Data file.

**Fig. 6:**
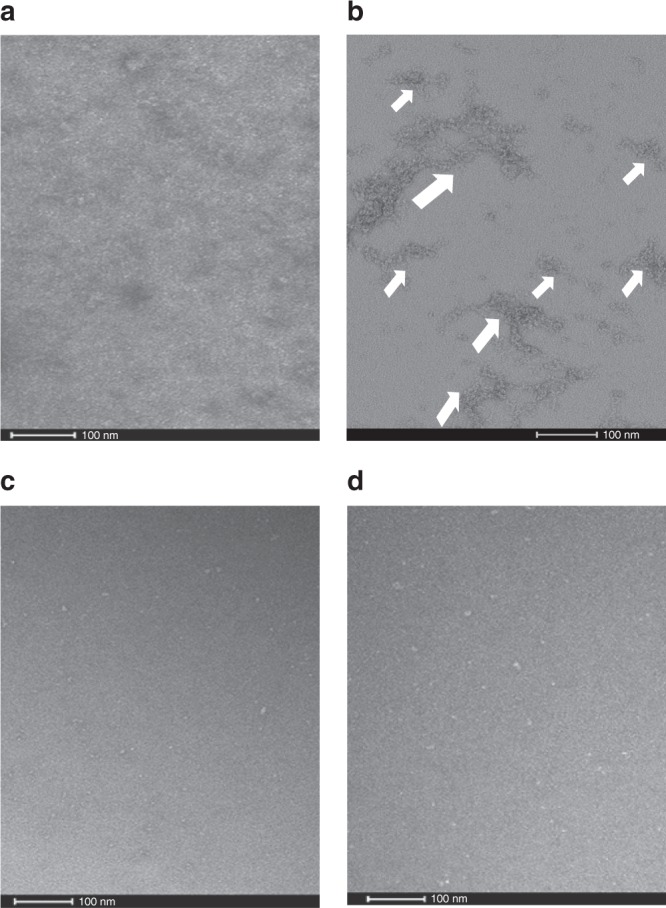
Effect of POA on PanD_WT_ and resistant mutant, non-POA binding PanD_127TRASC131_ as visualized by electron microscopy. Electron micrograph of negatively stained PanD_WT_ in the absence (**a**) and presence of 200 µM POA (**b**). The comparison visualizes oligomer formation upon drug binding to PanD_WT_. The different sized white arrows indicate the different sizes of oligomers formed. Control experiment showing PanD_127TRASC131_ mutant protein in the absence (**c**) and presence (**d**) of 200 µM POA, demonstrating that the formation of aggregates by POA is specific to the wild-type protein. Scale bars: 100 nm. These data show that POA causes oligomer formation of wild-type PanD but not of a POA-resistant mutant version of the protein. The experiment was carried out twice independently yielding the same results. These results are consistent with the corresponding results from dynamic light scattering experiments shown in Fig. 5.

**Fig. 7:**
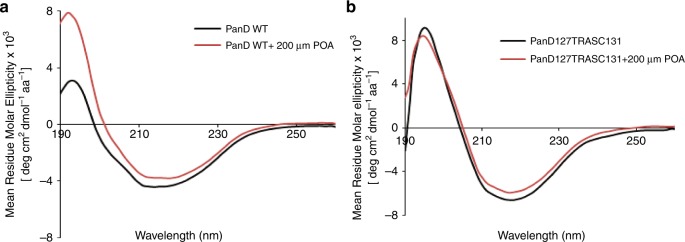
Effect of POA on PanD_WT_ and resistant mutant, non-POA binding PanD_127TRASC131_ as determined by circular dichroism spectroscopy. **a** Far UV CD spectra of PanD_WT_ without drug (black) and PanD_WT_ with 200 μM POA (red). The far UV spectrum of PanD_WT_ revealed a peak at 195 nm and a broad minimum at 218 nm indicative of a significant presence of β-sheets. The CD spectrum of drug-free PanD suggests a secondary structural content of 4% α-helices and 37.8% β-sheets, consistent with the secondary structural content determined by the crystallographic structure of PanD (PDB ID: 2C45). The presence of 200 μM POA caused an increase of mean residual molar ellipticity and decrease in breadth of the spectrum. **b** The far UV CD spectra of the PanD_127TRASC131_ mutant protein in the absence (black) and presence (red) of 200 µM POA. The CD spectra of the PanD_127TRASC131_ mutant in the presence and absence of 200 μM POA did not show the drastic effect at the 195 nm peak as observed for PanD_WT_ shown in **a**. No change in the broader range from 190 to 210 nm was observed for the mutant protein in the presence of the drug. Only a minor decrease of mean residual molar ellipticity was detected at the 218 nm minima in the mutant spectrum after addition of 200 μM POA. Two independent measurements were carried out and three spectra per sample were collected, yielding the same results. The results from a representative measurement are shown. These data show that POA causes a drastic change of the CD spectrum of wild-type PanD but has only minor effects on the CD spectrum of a POA-resistant mutant version of the protein. Source data are provided as a Source Data file.

Next, we resorted to biophysical methodologies to characterize the structural consequences of POA binding to PanD. In previous work, we have used small angle X-ray scattering (SAXS) analyses to show that PanD forms a monodispersed octamer in solution^4^. It is therefore possible that under drug-free conditions, PanD’s C-terminal tail is poorly accessible or even sequestered, resulting in a low degradation efficiency. Binding of POA may induce changes in the conformation and oligomeric state of PanD, rendering the protein’s degradation tag more accessible to the protease complex, thus allowing increased degradation. To determine whether POA indeed affects the oligomeric state of PanD, dynamic light scattering experiments were carried out. Under drug-free conditions, PanD showed one major peak corresponding to a hydrodynamic diameter of 8.9 ± 4.92 nm (Fig. 5). This molecule size corresponds to an octameric molecular weight of 105.6 ± 39.9 kDa and is thus consistent with the previous SAXS data. Addition of 200 μM POA resulted in the disappearance of the 8.9 nm peak and the emergence of four peaks with calculated hydrodynamic diameters of 21.04 ± 7.22, 55.04 ± 6.35, 955.4 ± 106.9, and 4801 ± 1134 nm (Fig. 5). The POA-induced formation of a series of larger complexes was visualized and confirmed by electron microscopy (Fig. 6). Together, these results show that POA binding causes the formation of a mixture of larger PanD ensembles and thus has a dramatic effect on the quaternary structure of the protein. To determine whether POA interaction with PanD also affects the protein’s secondary structural content, circular dichroism (CD) analyses were carried out (Fig. 7). The CD spectrum of drug-free PanD suggests a secondary structural content of 4% α-helices and 37.8% β-sheets, consistent with the secondary structural content determined by the crystallographic structure of PanD (PDB ID: 2C45). Addition of 200 μM POA caused a significant shift in the CD spectrum (Fig. 7), showing that POA binding alters the conformation of PanD. Taken together, these biophysical analyses show that POA binding causes major alterations at the secondary and quaternary structural levels. How these multilevel structural changes enhance protein degradation by ClpC1–ClpP remains to be determined.

## Discussion

We show here that POA, the bioactive component of PZA, blocks mycobacterial CoA biosynthesis by promoting degradation of its target PanD by the caseinolytic protease ClpC1–ClpP. Drug-induced degradation of a target protein is a novel antibacterial mechanism. However, this strategy does have precedents: selective estrogen receptor degraders, such as fulvestrant, used clinically for the treatment of certain breast cancers, are known to decrease intracellular estrogen receptor α levels^19^. In addition to fulvestrant, small-molecule phthalimides (e.g., thalidomide and lenalidomide)^20,21^ and the plant hormone Auxin^22^ were shown to induce the degradation of specific substrates. Interestingly, targeted protein degradation as a novel drug discovery approach has gained momentum in recent years^23^. PROTACs, heterobifunctional molecules which contain discrete binding moieties for the protein of interest and for E3 ligase, make use of the human ubiquitin—proteasome system to specifically degrade tagged proteins^24^. A first PROTAC drug is heading for clinical trials^25^. In conclusion, we report that an old tuberculosis drug acts by an event-based rather than occupation-based mechanism, accelerating degradation of its cellular target by Mtb’s own protease machinery.

## Methods

### Bacterial strains, culture medium, and chemicals

Mtb H37Rv (ATCC 27294) and *M. bovis* BCG (ATCC 35734) strains were maintained in complete Middlebrook 7H9 medium (BD Difco) supplemented with 0.05% (vol/vol) Tween 80, 0.5% (vol/vol) glycerol, 0.5% albumin, 0.2% glucose, 0.085% sodium chloride, and 0.0003% catalase at 310 K with agitation at 80 rpm. POA-resistant strain Mtb H37Rv POA^R^18 (*clpC1*: A625G/Lys209Glu) was isolated and described previously^7^. The Mtb H37Rv Δ*prcAB* strain (hygromycin-resistant) and its complemented mutant (hygromycin-resistant and Kanamycin-resistant) were previously generated and described^18^. Hygromycin B (Roche) and Kanamycin (Sigma-Aldrich) were used for selection when required at 50 and 25 µg/mL, respectively. PZA, POA, and Isoniazid were purchased from Sigma-Aldrich. Bortezomib was purchased from Chembridge. Antibiotics were dissolved in 90% DMSO and sterilized using 0.2 μm PTFE membrane filters (Acrodisc PALL).

### Susceptibility testing

MICs were performed by the broth dilution method as described^7^. The strains were grown to mid-log phase, spun down, resuspended in fresh 7H9 broth, and adjusted to an OD_600_ = 0.1. One hundred microliters of cell suspension was added into wells containing one hundred microliters twofold serially diluted compound in transparent flat-bottomed 96-well plates (Corning Costar), sealed with Breathe-Easy membranes (Sigma-Aldrich). The plates were incubated for 7 days at 310 K with shaking at 80 rpm. After incubation, the cultures were manually resuspended and OD_600_ was measured using a spectrophotometer (Tecan Infinite M200 Pro). Experiments were repeated at least once independently with two technical replicates. MIC_50_ values represent the concentration of drug which inhibits bacterial growth by 50% as compared with the respective drug-free control.

### Protein preparation

Wild-type Mtb Aspartate decarboxylase PanD (PanD_WT_) and the POA-resistant mutant PanD (PanD_127TRASC131_) proteins were overexpressed with N-terminal 6× His tags in *E. coli* and purified by Ni^2+^-NTA affinity, followed by gel filtration chromatography with buffer containing 50 mM Tris-HCl (pH 7.5), 200 mM NaCl as described previously^4^. Protein concentrations were determined using a BioSpec-nano spectrophotometer (Shimadzu, USA). Purity and homogeneity of samples were verified by SDS-PAGE (Fig. 1b).

### SDS-PAGE analyses of recombinant PanD

Protein samples were analyzed by 12% SDS-PAGE (NuPAGE Bis-Tris precast gel) run at a constant voltage of 200 V for 45 min in NuPAGE MES-SDS running buffer. The recombinant protein samples were denatured with a sample buffer containing LDS (NuPAGE), 0.1 M dithiothreitol (DTT) and heating at 368 K for 10 min prior to loading. The gels were stained with Coomassie Brilliant Blue R-250 (BioRad) as described by the suppliers.

### ^1^H-1D NMR studies of L-Asp to β-Ala conversion

NMR samples containing 2 mM L-Asp, 10 µM of PanD_WT_ or PanD_127TRASC131_ were prepared in deuterated water (D_2_O) buffer. NMR experiments were carried out using a Bruker Avance 400 MHz NMR spectrometer, equipped on a 5 mm BBI probe head at 298 K as described by Sharma et al.^26^. Basic ^1^H-1D NMR spectra were collected with presaturation of solvent water (relaxation delay 2 s; solvent presaturation applied during the relaxation delay). Time-dependent ^1^H-1D NMR experiments were performed to examine the kinetics of enzymatic conversion of L-Asp to β-Ala at six different incubation time points (0, 10, 20, 30, 40, 60, and 90 min) using PanD_WT_ (Fig. 1c, d). In order to study the inhibitory effect of POA, the compound was added (0.2 and 2 mM) to reaction mixtures, containing 2 mM of L-Asp. Overall, 10 µM of enzyme (PanD_WT_ or PanD_127TRASC131_) was added to initiate the reaction (spectra are shown in Supplementary Fig. 1).

### Fluorescence reporter strains and reporter assay

Genomic DNA was isolated from Mtb H37Rv (ATCC 27294), *M. bovis* BCG (ATCC 35734), POA-resistant strains *M. bovis* BCG POA1.3 (*panD*: A62G/His21Arg), and *M. bovis* BCG POA1.1 (*panD*: T407G/Leu136Arg)^6^. The sequences of *panD* in *M. bovis* BCG (ATCC 35734) and Mtb H37Rv (ATCC 27294) are 100% identical as determined by sequencing. Primers and templates used for plasmid construction of each RFP protein fusion are summarized in Supplementary Table 1. PCR amplification was performed with Phusion DNA polymerase (Thermo Scientific) as per the manufacturer’s instructions.

The obtained RFP fusion constructs were electroporated into Mtb H37Rv wild type, Mtb H37Rv POA^R^18 or wild-type *M. bovis* BCG as specified and selected on complete Middlebrook 7H11/7H10 agar containing Kanamycin (25 μg/mL) at 310 K. Kanamycin-resistant single colonies were picked and colony-purified. Strains were grown to log phase in respective growth media and stored in 7H9 broth containing 10% glycerol in 1 mL aliquots at 193 K. Strains derived from the above experiments are summarized in Supplementary Table 2.

For the reporter assay, the different fluorescent reporter strains were grown to mid-log phase (OD_600_ = 0.4–0.6) in their respective selective 7H9 media. All cultures were then adjusted to OD_600_ = 0.4 in fresh 7H9 broth after centrifugation at 3200 × rpm for 10 min and 100 µL of this suspension was inoculated into wells of a flat-bottomed 96-well plate (Costar Corning), each containing 100 µL of media (with or without drugs). Reporter assays were carried out with dual readout, absorbance (OD_600_), and relative fluorescence units (RFU; *λ*_ex_*/λ*_em_, 587/630 for RFP) by using an Infinite M200 Pro plate reader (Tecan). Baseline measurements at day 0 were carried out with RFU, after which plates were sealed with Breathe-Easy sealing membranes (Sigma-Aldrich) and incubated at 310 K with shaking at 80 rpm for measurements at subsequent time points.

### Cloning and synthesis of fluorescently tagged proteins

Constructs encoding eGFP alone, or eGFP fused to the N-terminus of proteins of interest were cloned into pET26b or pET21a to allow T7 polymerase initiated in vitro expression. Briefly, expression vectors were linearized by restriction digestion (NdeI and HindIII) and column purified. To make pET26b_eGFP, mycobacterial codon-optimized eGFP was amplified with JH-306 and JH-311, column purified, and cloned into linearized pET26b by Gibson assembly^27^. To generate eGFP fusion constructs, an 18 bp linker (5′-ggatctagcggatccagt-3′, encoding a flexible GSSGSS linker when being translated) was used as both the DNA homology for Gibson assembly and a flexible linker peptide. PCR amplified eGFP-linker (JH-306/JH-307) and linker protein of interest (Supplementary Table 1) were column purified and cloned into designated vectors using Gibson assembly.

In vitro protein expression was carried out following the manufacturer’s instructions (NEB, E6800s). Briefly, 300 ng of each column purified plasmid (Supplementary Table 1) was mixed with PURExpress solution A and B (10 and 7.5 µL, respectively), 0.5 µL of RNaseOUT^TM^ RNase inhibitors (Thermo), and nuclease-free water (Promega) to make final volume 25 µL. Protein synthesis was allowed by incubating the reaction mix at 310 K for 5 h, and then stopped by adding excessive amount of chloramphenicol (final concentration as 500 µg/mL). Successful protein production was validated by SDS-PAGE electrophoresis and Coomassie staining. The remaining products were kept at 277 K for no more than a week before further steps.

### In vitro reconstitution of PanD degradation

PanD degradation assays with purified recombinant ClpC1 and ClpP (ClpP1 and ClpP2) were carried out as described^17^. Full-length (eGFP_full_PanD) and N-terminally truncated (eGFP_PanD_ΔN-term1-24_, eGFP_PanD_ΔN-term1-24, ΔC-term127-139_) PanD proteins were produced with N-terminally fused eGFP in a cell-free transcription/translation system as described above. Aliquots from protein expression mixes were diluted fivefold into the proteolysis assay mixture containing 300 nM ClpP1P2 tetradecamer and 300 nM ClpC1 hexamer in reaction buffer (20 mM K-phosphate buffer pH 7.6 containing 100 mM KCl, 5% glycerol, 8 mM Mg-ATP, and 2.5 mM of dipeptide activator Bz-Leu-Leu). Degradation was followed by the loss of eGFP fluorescence^17^.

### Quantitative PCR

RNA from Mtb *H37Rv* wild type was isolated from an equivalent of 20 mL of culture at an OD_600_ = 0.4. Cultures were spun down at 3200 rpm for 10 min, resuspended in 1 mL TRIzol (Invitrogen), and subjected to bead beating by using a FastPrep-24 5G instrument (MP Biomedicals; twice for 45 s each, 5 min on ice between pulses). RNA was purified using the PureLink RNA mini kit with the Turbo DNA-free kit (Invitrogen) following the manufacturer’s instructions with on-column DNAse treatment (Invitrogen). cDNA was synthesized from 500 ng of total RNA with the SuperScript III first-strand synthesis system (Invitrogen) by using random primers (Promega). Quantitative PCR was performed using the FastStart Essential DNA Green Master (Roche) on a LightCycler 96 real-time PCR system (Roche) (for primers, see Supplementary Table 3). Relative expression of transcripts was determined by the ΔΔC_Q_ method as compared with 16S rRNA, which was uniformly expressed in our Mtb strains. cDNA was synthesized from at least two independent RNA samples and qRT-PCR was performed at least twice, in triplicate wells, for each cDNA sample.

### Preparation of mycobacterial cell lysates

*M. bovis* BCG wild type or mutant cultures were grown to mid-log phase in independent biological replicates and 100 mL of culture (adjusted to OD_600_ = 0.4) was harvested by centrifugation at 3400 × *g* for 20 min at 277 K, washed with ice-cold phosphate-buffered saline and pelleted again. For the western blotting experiments, mid-log cultures were adjusted to OD_600_ = 0.2 in fresh media and subjected to POA treatment (1 or 4 mM), the equivalent of 100 mL OD_600_ = 0.4 was harvested at specified time points. The cell pellets were resuspended in 600 µL lysis buffer (50 mM Tris/Hcl pH 7.5, 5% (vol/vol) glycerol, 1.5 mM MgCl_2_, 150 mM NaCl, 1 mM DTT, 1% n-dodecyl β-D-maltoside (w/vol), 1× complete EDTA-free protease inhibitor cocktail (Roche)), transferred to a lysis matrix B tube (MP Biomedicals) and homogenized using a FastPrep-24 5 G instrument (MP Biomedicals). The cell debris was pelleted by centrifugation at 13,000 × rpm for 10 min at 277 K and the supernatant (about 400 µL) was collected and stored at 193 K until further use. The protein concentration was determined by a BCA protein assay kit (Pierce).

### In-gel digestion

The protein lysates were concentrated by SpeedVac to 30 µL each. Samples were then polymerized with 4% SDS, 10% acrylamide (29:1 C), 0.25% ammonium persulfate, and 0.25% TEMED for 30 min. The gels were fixed with 50% methanol, 12% acetic acid for 30 min. The bands of interest were individually cut out from the gel (for protein identification from SDS-PAGE) or the entire polymerized cell lysates (prepared as described above) were cut into ~1 mm^3^ pieces and washed three times with 0.5 mL 50 mM triethylammonium bicarbonate buffer (TEAB), 50% (vol/vol) acetonitrile, and then treated with 500 µL acetonitrile. The gel pieces were reduced with 5 mM tris(2-carboxyethyl)phosphine (TCEP) in 100 mM TEAB and incubated at 329 K for 1 h. TCEP solution was removed and samples were dehydrated with acetonitrile. Solvent was removed, and samples were alkylated with 10 mM methyl methanethiosulfonate (MMTS) in 100 mM TEAB, followed by incubation at room temperature for 60 min. The gel pieces were washed with 50 mM TEAB and dehydrated with acetonitrile two times. The gel pieces were dried in a Speedvac. Overall, 12.5 ng/µL of Trypsin Gold (Promega) in 500 mM TEAB was added and kept at 277 K for 30 min. The mixture was incubated at 310 K for 16 h. Overall, 200 µL 50 mM TEAB was added to the digest, spun down at 6000 rpm for 10 min and the supernatant was collected. A total of 200 µL 5% formic acid (vol/vol), 50% acetonitrile was added to the gel pieces, spun down at 6000 rpm for 10 min and the supernatant was collected. The gel pieces were finally treated with 200 µL acetonitrile, followed by centrifugation at 6000 × rpm for 10 min. The three supernatants were combined and dried in a SpeedVac.

### LC–MS/MS analysis

The peptide separation was carried out on an Eksigent nanolC Ultra and ChiPLC-nanoflex (Eksigent, Dublin, CA, USA) in Trap Elute configuration. The samples were desalted with Sep-Pak tC 18 μ Elution Plate (Waters, Milford, MA, USA) and reconstituted with 20 μL of diluent (98% water, 2% acetonitrile, and 0.05% formic acid). Five microliters of the sample was loaded on a 200 μm × 0.5 mm trap column and eluted on an analytical 75 μm × 150 mm column. Both trap and analytical columns were made of ChromXP C18-CL, 3 μm (Eksigent, Germany). Peptides were separated by a gradient formed by 2% acetonitrile, 0.1% formic acid (mobile phase A) and 98% acetonitrile, 0.1% formic acid (mobile phase B): 5–7% of mobile phase B in 0.1 min, 7–30% of mobile phase B in 10 min, 30–60% of mobile phase B in 4 min, 60–90% of mobile phase B in 1 min, 90–90% of mobile phase B in 5 min, 90–5% of mobile phase B in 1 min, and 5–5% of mobile phase B in 10 min, at a flow rate of 300 nL/min.

The MS analysis was performed on a TripleTOF 5600 system (AB SCIEX, Foster City, CA, USA) in Information Dependent Mode. MS spectra were acquired across the mass range of 400–1250 *m/z* in high resolution mode (>30,000) using 250 ms accumulation time per spectrum. A maximum of 20 precursors per cycle were chosen for fragmentation from each MS spectrum with 100 ms minimum accumulation time for each precursor and dynamic exclusion for 8 s. Tandem mass spectra were recorded in high sensitivity mode (resolution >15,000) with rolling collision energy. Survey-IDA Experiment, with charge state 2–4, which exceeds 125 cps was selected.

Peptide identification was carried out on the ProteinPilot 5.0 software Revision 4769 (AB SCIEX) using the Paragon database search algorithm (5.0.0.4767) for peptide identification and the integrated false discovery rate analysis function. The data were searched against a database consisting of *M. bovis* BCG proteome UP000001472 (total 7782 entries). The search parameters are as follows: Sample type—identification; Cys alkylation—MMTS; digestion—trypsin; special factors—none; species—none. The processing was specified as follows: ID focus—biological modifications; search effort—thorough; detected protein threshold—0.05 (10.0%) and competitor Error Margin (ProtScore)—2.00.

### Western blot analyses of native PanD protein

Total protein extracts were prepared from *M. bovis* BCG cultures as described above and protein contents were quantified using the BCA protein assay kit (Pierce). Ten micrograms of total protein lysates was subjected to 12% SDS-PAGE (NuPAGE Bis-Tris precast gel and NuPAGE MES-SDS running buffer). Dual color markers (Biorad Precision Plus) and recombinant PanD_WT_ protein were included as molecular weight markers. Proteins were transferred onto 0.2 µm nitrocellulose membranes (Biorad) in a mini Trans-Blot cell (Biorad). Blots were blocked with 5% skimmed milk containing Phosphate-buffered saline with 0.1% Tween-20 (PBST) for 3 h at 298 K, and then probed with PanD-specific rabbit sera 1:500 in 5% skimmed milk-PBST overnight at 277 K. PanD-specific rabbit sera were obtained from i-DNA Technologies (Singapore) by immunizing rabbits with a synthetic peptide from the PanD aspartate decarboxylase domain (C-IAYATMDDARARTY-amide). Secondary HRP Goat-anti-rabbit antibodies (#65-6120 Invitrogen) were used at a 1:5000 dilution in 5% skimmed milk-PBST for 1 h at 298 K. Detection was performed with the Clarity Max Western ECL kit (Biorad) and visualized with a ChemiDoc imaging system (Biorad). To ensure equivalent loading of total protein lysates, blots were stripped and re-probed with Anti-RpoB monoclonal antibody (#12087 Abcam) at a 1:10,000 dilution in 5% skimmed milk-PBST and 1:20,000 secondary HRP Rabbit-anti-mouse antibody (#61–6520 Invitrogen) in 5% skimmed milk-PBST^16^. Unprocessed scans are available in the Source Data file.

### Dynamic light scattering studies

Dynamic light scattering experiments of PanD_WT_ and the mutant PanD_127TRASC131_ in the absence or presence of 200 µM POA were carried out using the Malvern Zetasizer Nano ZS spectrophotometer. The protein was incubated for 40 min with drug before measurement. The samples were measured in a low-volume quartz batch cuvette (ZEN2112, Malvern Instruments) using 12 µL of 1 mg/mL of PanD in 50 mM Tris (pH 7.5) buffer containing 200 mM NaCl. After 60 s equilibration time at 298 K, the backscattering at 173° was detected for all proteins. Scattering intensities were analyzed using the instrument software to calculate the hydrodynamic diameter (D_H_), size, and volume distribution.

### Electron microscopy

PanD_WT_ and the mutant PanD_127TRASC131_ was incubated with 200 μM POA for 40 min as described under dynamic light scattering before applying a volume of 4 μL of the drug-bound protein (50 μg/mL) to a glow discharged carbon-coated copper TEM grid and stained with 2% (v/v) uranyl acetate. Electron micrographs were recorded on a 120 kV Tecnai spirit T12 transmission electron microscope (FEI) equipped with a 4 K CCD camera (FEI) at a calibrated magnification of ×66,350 under low-dose conditions.

### Circular dichroism spectroscopy

CD spectra of PanD_WT_ and the mutant PanD_127TRASC131_ with or without 200 µM POA were recorded with a CHIRASCAN spectrometer (Applied Photo-physics) using a 60 µL quartz cell (Hellma, Germany) with 0.1 mm path length. The respective protein was incubated for 40 min with drug before measurement. Light of 190–260 nm was used to record the far UV–spectra at 298 K with 1 nm resolution. Two independent measurements were carried out and each measurement was performed three times for each sample. The CD spectra were acquired in a buffer of 50 mM Tris, pH 7.5, and 200 mM NaCl with protein concentration of 2.0 mg/mL. Secondary structural content was determined from CD spectrum using K2D3 software^28^.

### Reporting summary

Further information on research design is available in the Nature Research Reporting Summary linked to this article.

## Supplementary information

Supplementary Information

Peer Review File

Reporting Summary

Description of Additional Supplementary Files

Supplementary Data1

Supplementary Data 2

## Data Availability

The data that support this study are available from the corresponding author upon reasonable request. The source data underlying Figs. 1d, e, 2b, c, 3–5, and 7, and Supplementary Figs. 2–6 are provided as a source data file.

## Source data

Source Data
