# Supplementary material for: Pyrazinamide triggers degradation of its target aspartate decarboxylase
Source: Nat Commun. 2020 Apr 3;11:1661. doi: 10.1038/s41467-020-15516-1 (PMC7125159; doi:10.1038/s41467-020-15516-1)
Supplement: Supplementary file 1 — Supplementary Information [file 41467_2020_15516_MOESM1_ESM.pdf]

Supplementary Information

Pyrazinamide triggers degradation of its target aspartate decarboxylase

Gopal et al.

Supplementary Figures:

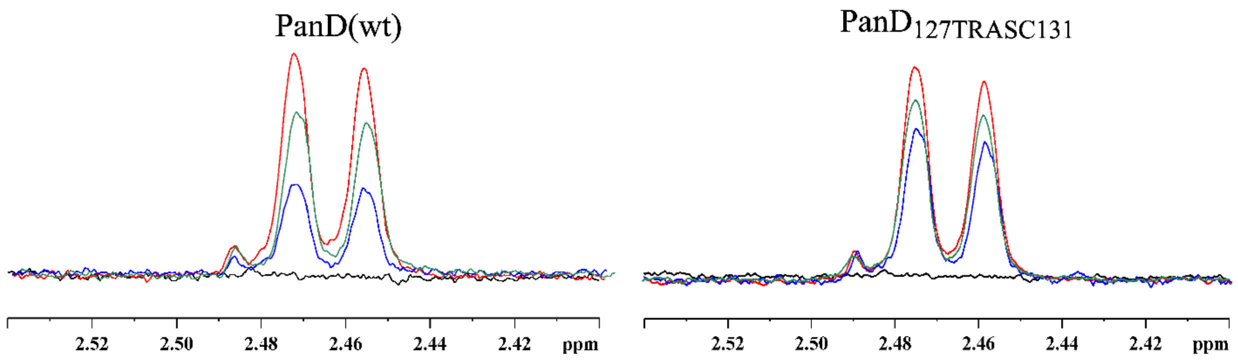

Supplementary Fig 1. Effect of POA on Mtb PanD enzyme activity as determined by ^{1}H NMR. Overlaid 1D NMR resonances of converted β-Alanine in $PanD_{WT}$ and POA-resistant C-terminal PanD mutant ($PanD_{127TRASC131}$) in the absence (*red*), and presence of 0.2 mM (*green*), or 2 mM (*blue*) POA after 40 min incubation at 298 K. The spectra in the absence of PanD enzymes are shown in black. The experiment was carried out two times independently. The results from a representative experiment are shown.

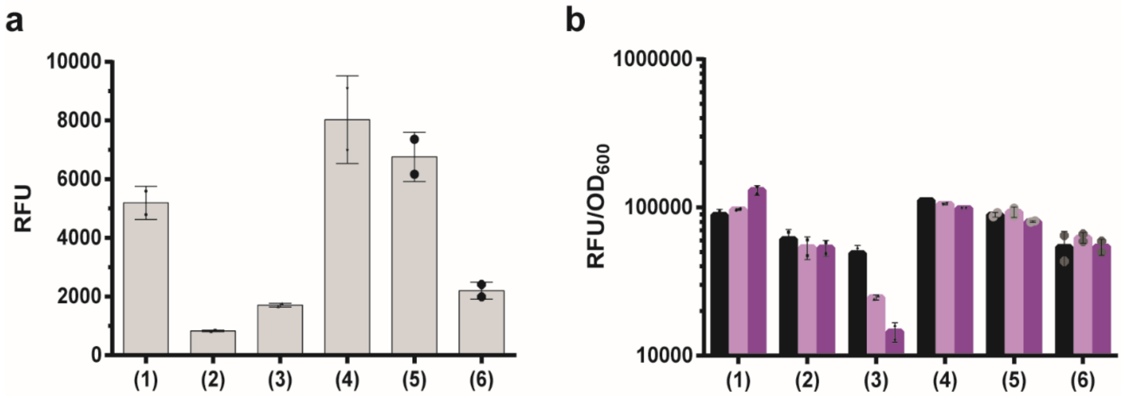

Supplementary Fig 2. Effect of POA treatment of *M. bovis* BCG on fluorescence levels of various PanD derivatives. (A) Drug-free fluorescence levels of mid-log phase cultures were determined as a measure of intra-bacterial fusion protein level in *M. bovis* BCG carrying RFP-PanD reporter constructs described in Fig. 2A. All reporter strains were grown to mid-log phase and adjusted to a final $OD_{600} = 0.2$. (*) indicate RFU means that were significantly different from fluorescence levels of the native RFP control (construct (1)) in *M. bovis* BCG background at p-value < 0.05, one-way ANOVA multiple comparisons test, GraphPad Prism. (B) *M. bovis* BCG harboring various PanD derivatives fused translationally to constitutively expressed red fluorescence protein (RFP) as reporter (Fig. 2A) were treated with increasing doses of POA (light purple, 1 mM; dark purple, 2 mM). See Fig. 2A for structure of RFP-PanD fusion constructs #1-6. For all experiments, fluorescence (expressed as RFU) was used as a measure of intra-bacterial fusion protein level normalized to bacterial growth (OD_{600}), measured after incubation for 3 days at 310 K. For each strain and treatment, drug-free controls were included (black bars). Experiments were carried out two times independently. Taken together, these data suggest that the mechanism of action of POA in *M. bovis* BCG is the same as observed in Mtb. Source data are provided as a source data file**.** Error bars were defined as standard deviations.

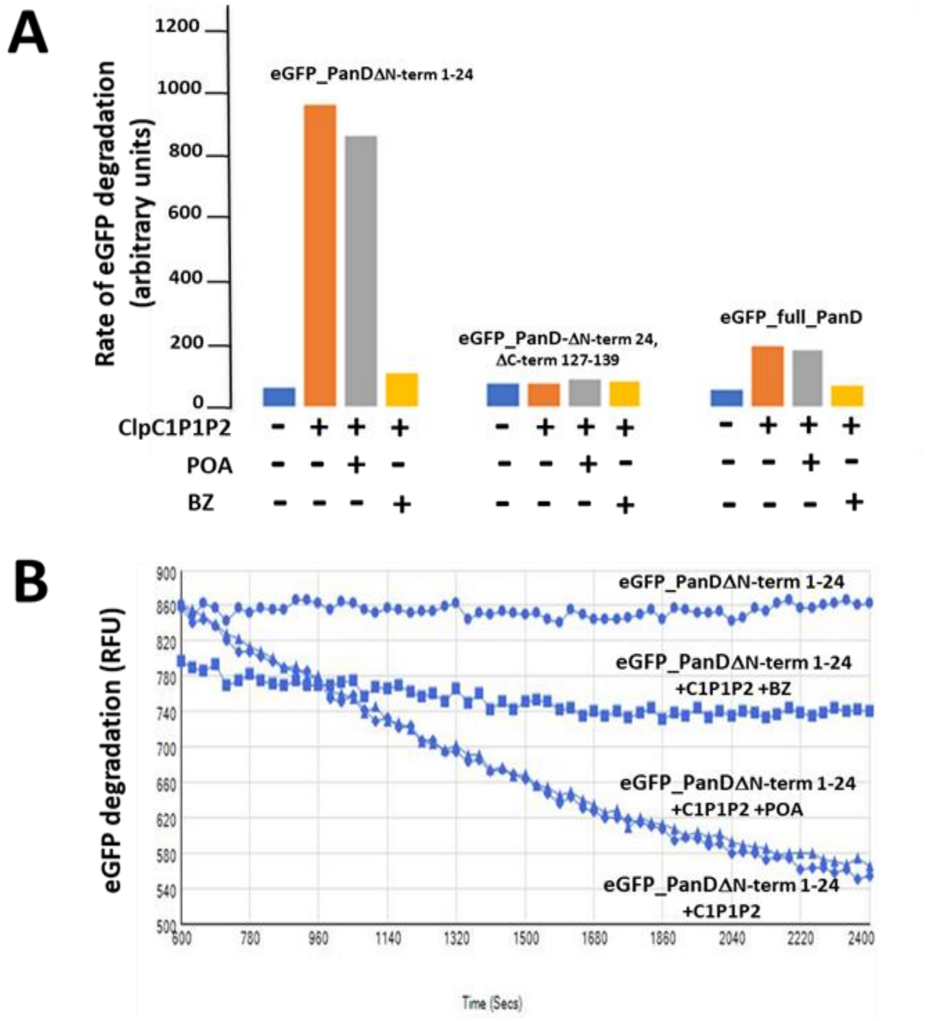

Supplementary Fig 3. *In vitro* degradation of PanD by ClpC1-ClpP. (A) N-terminal truncated PanD containing its C-terminal 13 amino acid degradation tag (eGFP_PanD$_{\Delta\text{N-term1-24}}$), N-terminally truncated PanD without its C-terminal degradation tag (eGFP_PanD $_{\Delta\text{N-term1-24, }\Delta\text{C-term127-139}}$) and full-length PanD (eGFP_full_PanD) were subjected to an *in vitro* ClpC1-ClpP degradation assay as described [1]. PanD proteins were generated in a cell-free translation system and harbored eGFP fused at their N-termini to enable measurement of protein levels over time. Addition of ClpC1-ClpP (ClpC1-ClpP1P2) caused a strong decrease of fluorescence (i.e. degradation) for eGFP_PanD $_{\Delta\text{N-term1-24}}$. The fluorescence level of eGFP_PanD $_{\Delta\text{N-term1-24, }\Delta\text{C-term127-139}}$, i.e. eGFP_PanD $_{\Delta\text{N-term1-24}}$ lacking the C-terminal degradation tag, was not affected by ClpC1-ClpP, demonstrating that degradation depends on PanD's C-terminal tail. Degradation of eGFP_PanD $_{\Delta\text{N-term1-24}}$ was suppressed by the ClpP inhibitor bortezomib (BZ) demonstrating that the loss of fluorescence is due to proteolysis. As expected, POA had no effect on the rate of degradation as this version of PanD lacks the His21 residue required for drug binding [2]. Fluorescence of full-length eGFP_full_PanD was not affected by ClpC1-ClpP. The reason for the apparent lack of recognition and degradation of the full-length protein by the protease complex remains to be determined. Possible explanations include for instance differences in the quaternary structure of

full-length PanD synthesized in a cell-free system when compared to protein produced *in vivo*. These differences may preclude access of the degradation tag by ClpC1-ClpP in the case of cell-free synthesized PanD. Additionally, auto-cleavage of full-length PanD may occur at Gly24-Ser25, potentially resulting in the detachment of reporter eGFP. In such a case, actual degradation would become non-detectable. BZ, bortezomib, concentration 100 µM; POA, pyrazinoic acid, concentration 1 mM. Source data are provided as a source data file. (B) Degradation of eGFP_PanD$_{\Delta N\text{-term}1\text{-}24}$ protein by ClpC1-ClpP in the absence or presence of bortezomib (BZ) or POA was followed continuously by measuring the change of eGFP fluorescence at 509 nm (ex 485 nm). RFU, relative fluorescence units. The experiments were carried out three times independently and a representative result is shown.

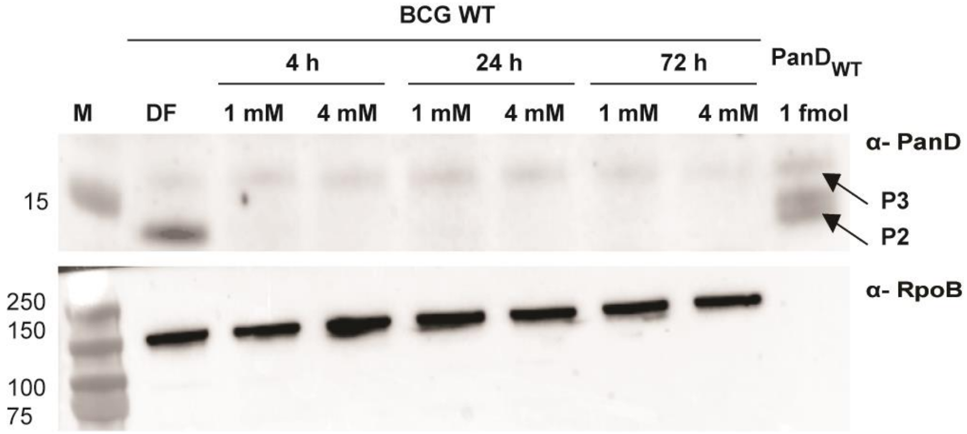

Supplementary Fig 4. Time kinetics of POA-induced PanD degradation in *M. bovis* BCG. *M. bovis* BCG wild-type (BCG WT) was treated with 1 mM or 4 mM POA and cells were harvested at 4 h, 24 h or 72 h after treatment for extraction of total protein. Drug-free (DF) controls were harvested at the start of the experiment (t = 0). 10 µg of respective total protein extracts were subjected to Western blot analyses. Upper panel: probing with α-PanD. $PanD_{WT}$, 1 fmol of recombinant PanD wild type protein was included as molecular weight marker. Lower panel: probing of blot showed in the upper panel with antiserum against mycobacterial RNA polymerase subunit RpoB (α-RpoB) to show equal loading. M, Marker with molecular weights (kilodalton) are indicated. Experiments were repeated 2 times independently and representative results are shown. These results show that POA treatment causes a rapid (4 h) and sustained (72 h) reduction of intra-bacterial PanD levels.

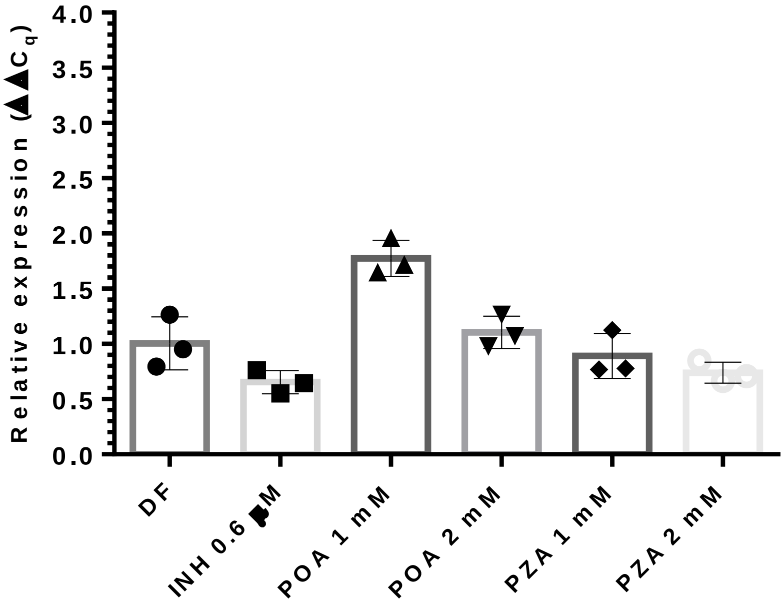

Supplementary Fig 5. *panD* mRNA levels in wild-type Mtb upon drug treatment. Transcript levels were measured from mid-log phase cultures (equivalent of 20 mL of OD_{600} = 0.4 were collected for each sample), adjusted to OD_{600} = 0.2 and incubated with or without drug for 24 h at 310 K. Drug-free (DF) controls were obtained at t = 0 h. INH 0.6 µM corresponds to 2 x Mtb MIC_{50}. Primer sequences can be found in Supplementary Table 3. Relative expression (quantification cycle [ΔΔCq]) was calculated as described previously [3] by using 16S RNA as the reference. The experiment was repeated two times independently with technical triplicates, yielding the same results. Mean and standard deviation (error bars) from technical triplicates of a representative experiment are shown. Means were found not to be significantly different from DF controls at p-value < 0.05 (*), one-way ANOVA multiple comparisons and Dunn's posttest, GraphPad Prism. These data show that treatment of bacteria with POA or PZA does not affect the mRNA level of *panD*. Source data are provided as a source data file.

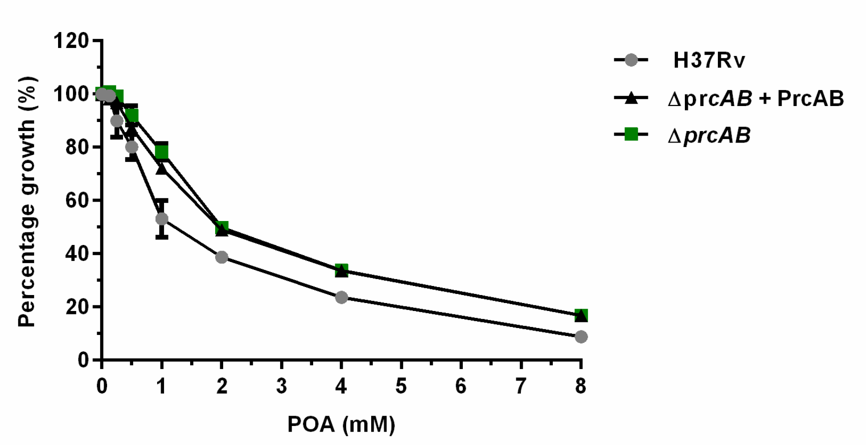

Supplementary Fig 6. Effect of deletion of the mycobacterial proteasome genes *prcAB* on the growth inhibitory activity of POA. The effect of increasing POA concentrations on growth inhibition of *M. tuberculosis* H37Rv, *M. tuberculosis* Δ*prcAB* and the *prcAB* complemented mutant strains [4] respectively. The experiment was carried out two times independently with technical duplicates. Mean and standard deviations (error bars) from a representative experiment are shown. These data suggest that the mycobacterial proteasome is not involved in the mechanism of action of POA. Source data are provided as a source data file.

Supplementary Tables

Supplementary Table 1. Fluorescent protein fusion plasmids and primers used in this study

Plasmid Name	Backbone plasmid (digested with)	Template DNA for PCR in this study	Inserted PCR-amplified DNA fragments		Reference (if any)
			Primer Name	Primer Sequence (5'->3')	
(1) RFP	pMV262 (BamHI-EcoRI)	Not applicable	mCh-F(BamHI)<br>mCh-R(EcoRI)	ccgggatccATGGTGAGCAAGGGCGAGG<br>ccggaattcCTACTTGTACAGCTCGTCCAT	*([5])*
(2) RFP-C-terminal	pMV262 (EcoRI-HindIII)	*Mtb* H37Rv	PanD FORWARD EcoRI_T<br>PanD REVERSE HindIII_T	ggaattcAACGCGGGCGAG<br>cccaagcttCTATCCCACACCG	This study
(3) RFP-PanD	pMV262 (EcoRI-HindIII)	*Mtb* H37Rv	PanD FORWARD EcoRI<br>PanD REVERSE HindIII	ccggaattcATGTTACGGACG<br>cccaagcttCTATCCCACACC	This study
(4) RFP-PanD no C-terminal	pMV262 (EcoRI-HindIII)	*Mtb* H37Rv	PanD FORWARD EcoRI<br>PanD w/o C-term REVERSE HindIII	ccggaattcATGTTACGGACG<br>cccaagcttCTATTCGGGCAC	This study
(6) RFP-PanD$_{\mathbf{L136R}}$	pMV262 (EcoRI-HindIII)	*M. bovis* BCG POA1.1*	PanD FORWARD EcoRI	ccggaattcATGTTACGGACG<br>cccaagcttCTATCCCACACC	This study

			PanD REVERSE HindIII		
(7) RFP-PanD$_{H21R}$	pMV262 (EcoRI-HindIII)	*M. bovis* BCG POA1.3[*]	PanD FORWARD EcoRI<br>PanD REVERSE HindIII	ccggaattcATGTTACGGACG<br>cccaagcttCTATCCCACACC	This study
pET26b_eGFP[!]	pET26b+ (NdeI-HindIII)	Not applicable	JH-306<br>JH-311	aactttaagaaggagatatacatatgtcgaagggcgaggagctg<br>tgctcgagtgcggccgcaagcttatttgtacagctcgtccatgccca	This study
pET26b_eGFP_ full_PanD[!]	pET26b+ (NdeI-HindIII)	*Mtb* H37Rv	JH-PAND_1<br>JH-PAND_2	tacaaaggatctagcggatccagtttacggacgatgctgaagtcg<br>tgctcgagtgcggccgcaagcttatcccacaccgagccggggg	This study
		pET26b_eGFP	JH-306<br>JH-307	aactttaagaaggagatatacatatgtcgaagggcgaggagctg<br>actggatccgctagatcctttgtacagctcgtccatgcc	
pET21a_eGFP_PanD$_{\Delta N\text{-}term1\text{-}24}$[#]	pET21a (NdeI-HindIII)	*Mtb* H37Rv	JH_PAND_11<br>JH-PAND_2	tacaaaggatctagcggatccagttcggtgaccatcgatgccg<br>tgctcgagtgcggccgcaagcttatcccacaccgagccggggg	This study
		pET26b_eGFP	JH-306<br>JH-307	aactttaagaaggagatatacatatgtcgaagggcgaggagctg<br>actggatccgctagatcctttgtacagctcgtccatgcc	
pET21a_eGFP_PanD$_{\Delta N\text{-}term1\text{-}24,\ C\text{-}term\ 127\text{-}139}$[#]	pET21a (NdeI-HindIII)	*Mtb* H37Rv	JH_PAND_11<br>JH-PAND_7	tacaaaggatctagcggatccagttcggtgaccatcgatgccg<br>tgctcgagtgcggccgcaagcttattcgggcacaaatgccggat	This study
		pET26b_eGFP	JH-306<br>JH-307	aactttaagaaggagatatacatatgtcgaagggcgaggagctg<br>actggatccgctagatcctttgtacagctcgtccatgcc	

[*] *M. bovis* BCG POA1.1 and *M. bovis* BCG POA1.3 were isolated and described in [6].

Numbered plasmids 1-7: used for *in vivo* studies. Non-numbered plasmids: used for *in vitro* translation.

[!] *in vitro* translation vector maintained in *E. coli* DH5α with Kanamycin selection.

[#] *in vitro* translation vector maintained in *E. coli* TOP10 with Ampicillin selection.

Supplementary Table 2. Bacterial strains used in this study

**Strain**	**Relevant Genotype/ Characteristics**	**Source**
M. tuberculosis **H37Rv (Mtb)**	wildtype	ATCC 27294
**Mtb RFP**	Fluorescent reporter with overexpression of (1) RFP (see Supplementary Table 1)	This study
Mtb RFP-C-terminal	Fluorescent reporter with overexpression of (2) RFP-C-terminal (see Supplementary Table 1)	This study
Mtb RFP-PanD	Fluorescent reporter with overexpression of (3) RFP-PanD (see Supplementary Table 1)	This study
Mtb RFP-PanD no C-terminal	Fluorescent reporter with overexpression of (4) RFP-PanD no C-terminal (see Supplementary Table 1)	This study
Mtb RFP-**PanD**$_{\mathbf{L136R}}$	Fluorescent reporter with overexpression of (3) RFP-PanD$_{\mathbf{L136R}}$ (see Supplementary Table 1)	This study
Mtb RFP-**PanD**$_{\mathbf{H21R}}$	Fluorescent reporter with overexpression of (3) RFP-PanD$_{\mathbf{H21R}}$ (see Supplementary Table 1)	This study
M. tuberculosis* Δ*prcAB	*ΔprcAB* obtained by homologous recombination following transduction with temperature-sensitive mycobacteriophage phAE87 that is ligated with modified plasmid pJSC284	([4])
M. tuberculosis Δ*prcAB* + prcAB	Complementation of prcAB in *ΔprcAB* strain using a mycobacterial plasmid containing the mycobacterial promoter P_{myc1}tetO and a kanamycin resistance gene	([4])
M. bovis **BCG** (BCG)	wildtype	ATCC 35734
BCG RFP	Fluorescent reporter with overexpression of (1) RFP (see Supplementary Table 1)	This study
BCG RFP-C-terminal	Fluorescent reporter with overexpression of (2) RFP-C-terminal (see Supplementary Table 1)	This study
BCG RFP-PanD	Fluorescent reporter with overexpression of (3) RFP-PanD (see Supplementary Table 1)	This study

BCG RFP-PanD no C-terminal	Fluorescent reporter with overexpression of (4) RFP-PanD no C-terminal (see Supplementary Table 1)	This study
BCG RFP- $PanD_{L136R}$	Fluorescent reporter with overexpression of (3) RFP-$PanD_{\mathbf{L136R}}$ (see Supplementary Table 1)	This study
BCG RFP- $PanD_{H21R}$	Fluorescent reporter with overexpression of (3) RFP-$PanD_{\mathbf{H21R}}$ (see Supplementary Table 1)	This study
E. coli BL21 (DE3)	Used for protein production	Stratagene

Supplementary Table 3. Primers used for qRT-PCR

**Target**	**Primer**	**Primer sequence**	**Amplicon (bp)**	**Reference**
16S	Fwd<br>Rev	ATGACGGCCTTCGGGTTGTAA<br>CGGCTGCTGGCACGTAGTTG	160	*([5])*
panD	Fwd<br>Rev	TACGGACGATGCTGAAGTCG<br>CGATGGTTACCTGTTCGCCT	135	This study

Supplementary References:

1. Akopian T*, et al.* Cleavage Specificity of Mycobacterium tuberculosis ClpP1P2 Protease and Identification of Novel Peptide Substrates and Boronate Inhibitors with Anti-bacterial Activity. *Journal of Biological Chemistry* **290**, 11008-11020 (2015).

2. Gopal P*, et al.* Pyrazinoic Acid Inhibits Mycobacterial Coenzyme A Biosynthesis by Binding to Aspartate Decarboxylase PanD. *ACS Infectious Diseases* **3**, 807-819 (2017).

3. Pfaffl MW. A new mathematical model for relative quantification in real-time RT–PCR. *Nucleic Acids Research* **29**, e45-e45 (2001).

4. Gandotra S, Lebron MB, Ehrt S. The Mycobacterium tuberculosis Proteasome Active Site Threonine Is Essential for Persistence Yet Dispensable for Replication and Resistance to Nitric Oxide. *PLOS Pathogens* **6**, e1001040 (2010).

5. Yamada Y, Dick T. Mycobacterial Caseinolytic Protease Gene Regulator ClgR Is a Substrate of Caseinolytic Protease. *mSphere* **2**, (2017).

6. Gopal P*, et al.* Pyrazinamide Resistance Is Caused by Two Distinct Mechanisms: Prevention of Coenzyme A Depletion and Loss of Virulence Factor Synthesis. *ACS Infectious Diseases* **2**, 616-626 (2016).
