# Supplementary material for: Pyrazinamide triggers degradation of its target aspartate decarboxylase
Source: Nat Commun. 2020 Apr 3;11:1661. doi: 10.1038/s41467-020-15516-1 (PMC7125159; doi:10.1038/s41467-020-15516-1)
Supplement: Supplementary file 4 — Description of Additional Supplementary Files [file 41467_2020_15516_MOESM4_ESM.docx]

**Description of Additional Supplementary Files**

File name: Supplementary Data 1

Description: Distinct peptide summary for peptides identified from the gel bands of recombinant Aspartate decarboxylase PanDWT. P1, P2 and P3 were gel bands cut out from an SDS-PAGE analysis as shown in Fig. 1. Shown are distinct peptide sequences and modifications identified from database search of Mtb Aspartate decarboxylase PanD (Rv3601c, TB database; P9WIL3, Uniprot).

File name: Supplementary Data 2

Description: Complete list of proteins identified through proteomic analysis of M. bovis BCG wild-type. List of all identified proteins for three independent biological replicates with the following data included: Percent of protein sequence covered, Uniprot accession number, Protein definition, Number of peptides identified. Members of the coenzyme A biosynthetic pathway are highlighted in red.
