# Supplementary material for: Pyrazinamide triggers degradation of its target aspartate decarboxylase
Source: Nat Commun. 2020 Apr 3;11:1661. doi: 10.1038/s41467-020-15516-1 (PMC7125159; doi:10.1038/s41467-020-15516-1)
Supplement: Supplementary file 2 — Peer Review File [file 41467_2020_15516_MOESM2_ESM.pdf]

REVIEWERS' COMMENTS:

Reviewer #1 (Remarks to the Author):

In this interesting and sound study, Gopal et al. investigate the mechanism of action of pyrazinamide - a drug used for the treatment of tuberculosis. The authors and others had previously shown that aspartate decarboxylase, an enzyme involved in pantothenate biosynthesis and encoded by the PanD gene, is the target of the drug – and identified drug-resistance mutations in both PanD and genes encoding the degrading protease complex ClpC1-ClpP. However, the exact mechanism of action remained elusive.

Here, the authors provide convincing evidence in support of a mode of action by which the drug works by inducing ClpC1-ClpP protease-mediated recognition and degradation of PanD in MtB cells. The paper provides evidence that supports the C-terminal tail of the PanD protein as a "degron" for ClpC1-ClpP mediated degradation. Using a constitutively-expressed fluorescence-fusion reporter assay, they show that addition of pyrazinamide enhances the degradation of the fluorescence fusion protein in cells and that this degradation is not observed in the presence of drug-resistant mutations. The authors then also show POA-induced degradation of the endogenous protein, supporting the proposed mechanism.

The elucidation that pyrazinamide works as a degrader of its target protein as the mode of action for its anti-tubercular activity is an important and novel finding. It is also unprecedented for antimicrobial drugs and will no doubt motivate more research in this direction. I therefore feel this discovery has the significance and timeliness to warrant publication in Nature Commun.

My only minor comment is that there were only 2 figures in the main text? The data showing POA-induced degradation of endogenous PanD seems sufficiently important imho so the authors might want to consider displaying it in the main text to avoid it getting lost in the Supporting Information.

Sincerely, Alessio Ciulli

Reviewer #2 (Remarks to the Author):

In their submitted manuscript, Gopal et al provide clear and convincing evidence that resolves longstanding questions about the mechanism of action of pyrazinamide. This work should be of considerable interest to biomedical scientists since pyrazinamide is standard of care for Mtb, which infects two BILLION people worldwide and is becoming increasingly drug resistant. Detailed knowledge of the elusive mechanism of action of pyrazinamide increases the likelihood of developing new and improved therapeutics with efficacy against resistant Mtb strains.

Minor editing suggestions:
A superscripted number is an awkward noun. If you remove the word "in" from "...as described in (ref)" (eg lines 324, 326, 333, 374), the sentence ends "...as described (ref)" and the reference functions merely as a reference.

Congratulations on your fascinating and important discovery, and thank you for your elegant demonstration of this novel antibiotic mechanism!

Charles P. Scott, Ph.D.
Thomas Jefferson University

Reviewer #3 (Remarks to the Author):

The mechanism of action of Pyrazinamide has been elusive due to the complex phenotypes of mutants. This paper provides a novel mechanism of drug action and demonstrates that Pyrazinoic acid targets the carboxy terminus of the panD-encoded aspartate decarboxylase by stimulating a cellular protease, which is required for Co-A synthetase. The authors had previously discovered that resistance to Pyrazinoic acid maps to specific regions of the panD enzyme as well as the gene encoding ClpD proteases. In this study, the authors have used constructs of an RFP fused to the carboxy terminus of panD to demonstrate that the Clp protease degrades the carboxy tagged RFP and not the parental RFP. This study convincingly demonstrates a novel mechanism of anti-bacterial action for pyrazinamide. The paper is well written and clearly provides a novel, and unexpected mechanism of action. Despite these advances, there are still unanswered questions that the authors need to address in their discussion.

1) Pyrazinamide, in combination INH and RIF, is known to rapidly sterilize Mtb infections yet pantothenate auxotrophs are not rapidly cleared in mice, including immunocompromised mice. The authors should suggest an explanation in the discussion.
2) The authors have not addressed the point about pH. Do they find a difference in the degradation of the panD carboxy terminal constructs if the pH is varied in their assays?
3) The authors should provide a list of the other Mtb proteins that shares the carboxy terminus with this aspartate decarboxylase. This could explain why the pantothenate auxotrophs are not rapidly cleared.

Minor Point:
-Please include a strain table that has the strain name, genotype, how constructed, and reference. This will facilitate the reading of the papers as well as provide specifics for other researchers that may want to use these strains.

Point-by-point reply to reviewers' comments:

REVIEWERS' COMMENTS:

Reviewer #1 (Remarks to the Author):

In this interesting and sound study, Gopal et al. investigate the mechanism of action of pyrazinamide - a drug used for the treatment of tuberculosis. The authors and others had previously shown that aspartate decarboxylase, an enzyme involved in pantothenate biosynthesis and encoded by the PanD gene, is the target of the drug – and identified drug-resistance mutations in both PanD and genes encoding the degrading protease complex ClpC1-ClpP. However, the exact mechanism of action remained elusive.

Here, the authors provide convincing evidence in support of a mode of action by which the drug works by inducing ClpC1-ClpP protease-mediated recognition and degradation of PanD in MtB cells. The paper provides evidence that supports the C-terminal tail of the PanD protein as a "degron" for ClpC1-ClpP mediated degradation. Using a constitutively-expressed fluorescence-fusion reporter assay, they show that addition of pyrazinamide enhances the degradation of the fluorescence fusion protein in cells and that this degradation is not observed in the presence of drug-resistant mutations. The authors then also show POA-induced degradation of the endogenous protein, supporting the proposed mechanism.

The elucidation that pyrazinamide works as a degrader of its target protein as the mode of action for its anti-tubercular activity is an important and novel finding. It is also unprecedented for antimicrobial drugs and will no doubt motivate more research in this direction. I therefore feel this discovery has the significance and timeliness to warrant publication in Nature Commun.

My only minor comment is that there were only 2 figures in the main text? The data showing POA-induced degradation of endogenous PanD seems sufficiently important imho so the authors might want to consider displaying it in the main text to avoid it getting lost in the Supporting Information.

Sincerely, Alessio Ciulli

Authors: We transferred the figure showing POA-induced degradation of endogenous PanD as well as several other relevant figures from supplementary materials to the main text. We would like to thank Dr. Ciulli for his comments.

Reviewer #2 (Remarks to the Author):

In their submitted manuscript, Gopal et al provide clear and convincing evidence that resolves longstanding questions about the mechanism of action of pyrazinamide. This work should be of considerable interest to biomedical scientists since pyrazinamide is standard of care for Mtb, which infects two BILLION people worldwide and is becoming increasingly drug resistant. Detailed knowledge of the elusive mechanism of action of pyrazinamide increases the likelihood of developing new and

improved therapeutics with efficacy against resistant Mtb strains.

Minor editing suggestions:
A superscripted number is an awkward noun. If you remove the word "in" from "...as described in (ref)" (eg lines 324, 326, 333, 374), the sentence ends "...as described (ref)" and the reference functions merely as a reference.

Congratulations on your fascinating and important discovery, and thank you for your elegant demonstration of this novel antibiotic mechanism!

Charles P. Scott, Ph.D.
Thomas Jefferson University

Authors: Agree. Superscripted numbers as nouns look indeed awkward. We changed these parts accordingly and deleted 'in'. We would like to thank Dr. Scott for his comments.

Reviewer #3 (Remarks to the Author):

The mechanism of action of Pyrazinamide has been elusive due to the complex phenotypes of mutants. This paper provides a novel mechanism of drug action and demonstrates that Pyrazinoic acid targets the carboxy terminus of the panD-encoded aspartate decarboxylase by stimulating a cellular protease, which is required for Co-A synthetase. The authors had previously discovered that resistance to Pyrazinoic acid maps to specific regions of the panD enzyme as well as the gene encoding ClpD proteases. In this study, the authors have used constructs of an RFP fused to the carboxy terminus of panD to demonstrate that the Clp protease degrades the carboxy tagged RFP and not the parental RFP. This study convincingly demonstrates a novel mechanism of anti-bacterial action for pyrazinamide. The paper is well written and clearly provides a novel, and unexpected mechanism of action. Despite these advances, there are still unanswered questions that the authors need to address in their discussion.

1) Pyrazinamide, in combination INH and RIF, is known to rapidly sterilize Mtb infections yet pantothenate auxotrophs are not rapidly cleared in mice, including immunocompromised mice. The authors should suggest an explanation in the discussion.

Authors: Good point. As we discussed recently (Gopal et al.; Pharmacological and Molecular Mechanisms Behind the Sterilizing Activity of Pyrazinamide. Trends in Pharmacological Sciences, 2019, 40, 12 https://doi.org/10.1016/j.tips.2019.10.005) it is likely that this fragment-sized drug has additional targets, other than PanD. Thus, PZA may employ poly-pharmacology to exert its anti-bacterial activity. This would explain the stronger bactericidal effect of the drug when compared to the phenotype of pantothenate auxotrophs. As this aspect was discussed in a recent opinion article we would prefer not to engage again in this discussion in this PanD-mechanism focused manuscript. We hope this is acceptable.

2) The authors have not addressed the point about pH. Do they find a difference in the degradation of the panD carboxy terminal constructs if the pH is varied in their assays?

Authors: We have not determined pH dependence of degradation. We could only isolate POA resistant Mtb mutants on 'neutral' pH agar. Selection attempts on acidic pH agar did not yield any mutants (Gopal et al.; Pyrazinamide Resistance Is Caused by Two Distinct Mechanisms: Prevention of Coenzyme A Depletion and Loss of Virulence Factor Synthesis. ACS Infect. Dis. 2016, 2, 9, 616-626, https://doi.org/10.1021/acsinfecdis.6b00070). Accordingly, we observed that resistance is only phenotypically expressed when mutant strains are grown in neutral pH broth. Thus, it appears that the mechanisms under investigation are only operative under neutral pH. Therefore, we have not investigated pH dependence of PanD degradation.

3) The authors should provide a list of the other Mtb proteins that shares the carboxy terminus with this aspartate decarboxylase. This could explain why the pantothenate auxotrophs are not rapidly cleared.

Authors: There are only few recognition tags for Clp identified and they do not show a 'consensus' sequence (see for instance Yamada, Dick; Mycobacterial Caseinolytic Protease Gene Regulator ClgR Is a Substrate of Caseinolytic Protease. mSphere. 2017;2(2):e00338-16. doi: 10.1128/mSphere.00338-16). We did carry out a BLAST search in an attempt to identify protein with C-terminal sequences similar to the mycobacterium-specifc C-terminal extension of PanD - but did not find convincing hits. Thus, we can't provide a list of other Mtb proteins that share PanD's C-terminus.

Minor Point:

-Please include a strain table that has the strain name, genotype, how constructed, and reference. This will facilitate the reading of the papers as well as provide specifics for other researchers that may want to use these strains.

Authors: we added a strain table containing the suggested information to the Supplementary Information. We would like to thank reviewer #3 for her/his comments.
